# Clinical simulation with dramatization: gains perceived by students and health professionals

**DOI:** 10.1590/1518-8345.1807.2916

**Published:** 2017-08-03

**Authors:** Elaine Cristina Negri, Alessandra Mazzo, José Carlos Amado Martins, Gerson Alves Pereira, Rodrigo Guimarães dos Santos Almeida, César Eduardo Pedersoli

**Affiliations:** 1 Doctoral student, Escola de Enfermagem de Ribeirão Preto, Universidade de São Paulo, PAHO/WHO Collaborating Centre for Nursing Research Development, Ribeirão Preto, SP, Brazil.; 2 PhD, Associate Professor, Escola de Enfermagem de Ribeirão Preto, Universidade de São Paulo, PAHO/WHO Collaborating Centre for Nursing Research Development, Ribeirão Preto, SP, Brazil.; 3 PhD, Professor, Escola Superior de Enfermagem de Coimbra, Coimbra, Portugal.; 4 PhD, Professor, Faculdade de Medicina de Ribeirão Preto, Universidade de São Paulo, Ribeirão Preto, SP, Brazil.; 5 PhD, Adjunct Professor, Universidade de Ribeirão Preto, Ribeirão Preto, SP, Brazil.

**Keywords:** Students, Role Playing, Patient Simulation, Education, Perception

## Abstract

**Objective:**

to identify in the literature the gains health students and professionals perceive when using clinical simulation with dramatization resources.

**Method:**

integrative literature review, using the method proposed by the Joanna Briggs Institute (JBI). A search was undertaken in the following databases: Latin American and Caribbean Health Sciences Literature, Web of Science, National Library of Medicine, Cumulative Index to Nursing and Allied Health Literature, The Cochrane Library, Scopus, Scientific Electronic Library Online.

**Results:**

53 studies were analyzed, which complied with the established inclusion criteria. Among the different gains obtained, satisfaction, self-confidence, knowledge, empathy, realism, reduced level of anxiety, comfort, communication, motivation, capacity for reflection and critical thinking and teamwork stand out.

**Conclusion:**

the evidence demonstrates the great possibilities to use dramatization in the context of clinical simulation, with gains in the different health areas, as well as interprofessional gains.

## Introduction

As a result of the needs of a globalized society, immersed in Information and Communication Technologies (ICT) and going through a continuous process of scientific and technological modernization, teaching in health and nursing has undergone transformations, adapting competencies, critical thinking and decision making skills^1^ ^-^ ^3^.

To satisfy these needs, the professional education underwent restructuring, which has slowly provoked the evolution of knowledge and complex thinking, aiming to prepare more critical and reflexive professionals, capable of acting in a wide range of situations. In that context, the teaching institutions have reconsidered the educational practices and employed innovative strategies, with a view to stimulating competent professionals, which has highlighted the use of clinical simulation as a necessary and valued tool in the teaching-learning process^1^ ^-^ ^4^.

The act of teaching through clinical simulation has frequently been part of the undergraduate curriculum, and also of health professionals’ training. Nevertheless, as a result of the advances in the structuring of the strategy and the increased capacity to gain competencies, critical reasoning, decision making and teamwork and to strengthen the professionals’ self-confidence, it has been increasingly valued and enhanced as a teaching strategy^5^ ^-^ ^8^.

In simulated clinical practice, several resources can be used, ranging from dramatization to the use of inanimate anatomic pieces and/or advanced simulators, which incorporate high computer and robotic technology and lead to many interaction possibilities, with great variation in the costs involved. In the construction of the simulated scenarios, physical and material resources are employed that approach the actual activities of clinical practice involving patients with a high degree of realism. The resources are defined according to the learning objectives and are classified according to their technological potentials^6^ ^,^ ^9^.

Among the resources applied in this study, the dramatization technique will be highlighted. Dramatization can be defined as a theatre representation, determined based on a focus or theme. This resource grants meanings and permits the contents taught to be experienced in a context similar to those experienced in the actual practice^10^. Dramatization allows the student to integrate theory and practice, it is flexible and adjustable to different contexts, permits experiencing different perspectives and viewpoints and offers the student the opportunity to explore the individual vulnerability in a safe environment^11^.

In dramatization, the techniques explored can be role play and the use of simulated patients, mixed models and standardized patients.

Role play is the situation in which the learner, facilitator and/or instructor play different roles in the simulated scenario as if they were taking part in a clinical case, for the purpose of teaching and training^10^. This strategy grants learning opportunities, involving both the student’s affective and cognitive process, as they permit experiencing feelings, such as the experience of the patient’s and other professionals’ roles^12^.

Educators and clinical simulation researchers frequently use the expressions “simulated patient” and “standardized patient” interchangeably or as synonyms in the literature, although differences exist between them. Simulated patients are trained individuals and/or actors who play a role, exhibiting a story within the simulation for the purpose of teaching or assessment^13^.

The term standardized patient can be defined as: a member of the community (child, adolescent, adult, elderly) who agreed to play the role of a patient for a learning activity, through a legal contract with the teaching institution. The standardized patients do not play a role to perform the characteristics of another person or patient, but they answer any inquiry about the medical and social history based on their own lives^13^. This resource has served as a concrete possibility to provide clinical skills teaching and training, in function of its potential to comply with conditions closer to the ideal, guaranteeing the reliability of human interaction with communication and empathy^10^. For ethical and legal reasons, this technique has not been much used in Brazil^14^.

The mixed models enable the learner to develop technical and behavioral skills. They combine the simulated patient with a low-fidelity simulator to develop a specific activity in a scenario, such as an arm coupled to a student in a blood collection scenario for example^14^ ^-^ ^15^.

Due to its reasonable cost and great application possibility, the use of simulated practices with dramatization resources can turn into an excellent ally for the qualification of professionals with critical and reflexive thinking, who are capable of reaching clinical judgments and making decisions. Nevertheless, to better use the technique, its use should be based on scientific evidences that demonstrate the positive or negative results of this teaching and learning strategy.

In that context, to better understand and employ the available resources related to the theme, the objective in this study was to identify, in the literature, the gains the health students and professionals perceived in the use of clinical simulation with dramatization resources.

## Method

An integrative review was undertaken, using the method of the Joanna Briggs Institute (JBI), which is focused on the feasibility, adequacy, significance and efficacy of the health interventions. This method can be used to map the main concepts that sustain a research area, as well as to clarify the operational definitions and/or conceptual limits of a topic^16^.

To construct the research question, the PICO strategy was used in the quantitative articles: P - Students and professionals; I - Clinical simulation using dramatization; O - Perceived gains from clinical simulation using dramatization; and PICo in the qualitative articles PICo: P - Students and professionals; I - Clinical simulation and dramatization; Co - Perceived gains from clinical simulation using dramatization^17^.

This strategy permitted formulating the following guiding question: *What are the gains the health students and professionals perceive from the use of clinical simulation with dramatization resources?*

Thus, after establishing the question, an initial search was undertaken in the portal PubMed (Public Medline) and in the database CINAHL (Cumulative Index to Nursing and Allied Health Literature), in order to identify the main descriptors and key words used in the studies that discussed the theme of interest in this review.

To answer the research question, the controlled and non-controlled descriptors were selected, related to each of the components of the PICO and PICo strategy, used according to the Health Sciences Descriptors (DEsCS) and Medical Subject Headings (MeSH).

The research was developed between June and December 2015 without any restrictions in terms of time, presentation or publication type, using the following controlled descriptors: Students; Role Playing; Patient Simulation; Education; Perception; and the non-controlled descriptors: Professional; Patients Standardized; Standardized Patient; Dramatization; Clinical Simulation; Experience. In between the descriptors, the following Boolean operators were considered: *Students* AND *Professional* AND *Role Playing* OR *Patient Simulation* OR *Patients Standardized* OR *Standardized Patient* OR *Dramatization* OR *Clinical Simulation* AND *Education* OR *Perception* OR *Experience.*

Inclusion and exclusion criteria were established for the research, considering a number of study types: 1) studies involving health students and professionals; 2) studies that discussed the theme simulation with dramatization, that is, role play, standardized patients, patient simulation, mixed patient; 3) studies with a quantitative and/or qualitative focus, which answered the question established, independently of the knowledge area they were linked to and 4) studies published in Portuguese, English and Spanish. Publications of opinions, consensus statements, retractions, editorials and experience reports were excluded.

To identify the studies, the following electronic databases were used: Latin American and Caribbean Health Sciences Literature (LILACS), Web of Science, National Library of Medicine (PubMed), Cumulative Index to Nursing and Allied Health Literature (CINAHL), The Cochrane Library, Scopus, Scientific Electronic Library Online (SciELO).

In total, 6,826 studies were found, which were moved to Web ENDNOTE. Of these, 1,414 were excluded because the studies had been published in more than one database, resulting in 5,412 studies. After reading the titles and abstracts of the 5,412 research articles, 5,103 were excluded because they did not answer the research question and 309 were selected to read the full article. Among the 309 studies analyzed, 53 were included in the research because they answered the question and because they complied with the inclusion criteria established.

Next, the research data were analyzed with the help of a tool the researchers had constructed, in accordance with the JBI instructions^16^, including: study title, authorship, journal, year of publication, place of study (country), research objective(s), methodological details, sample details, main outcomes and conclusions found. In the critical analysis of the selected articles, the research design was analyzed^18^.

## Results

Among the 53 (100%) studies in the sample, the majority had been published in English. The studies had been mostly developed on the American (n=27, 50.94%), Asian (n=9, 17.0%), Oceania (n=9, 17.0%) and European continents (n=8, 15.1%).

When the type of dramatization the studies employed was analyzed, it was verified that 28 (52.9%) used a simulated patient; 18 (34.0%) role play; 4 (7.5%) dramatization with standardized patient; 2 (3.7%) simulated patient plus role play and 1 (1.9%) mixed patient (simulated patient plus pelvis).

**Figure 1:**
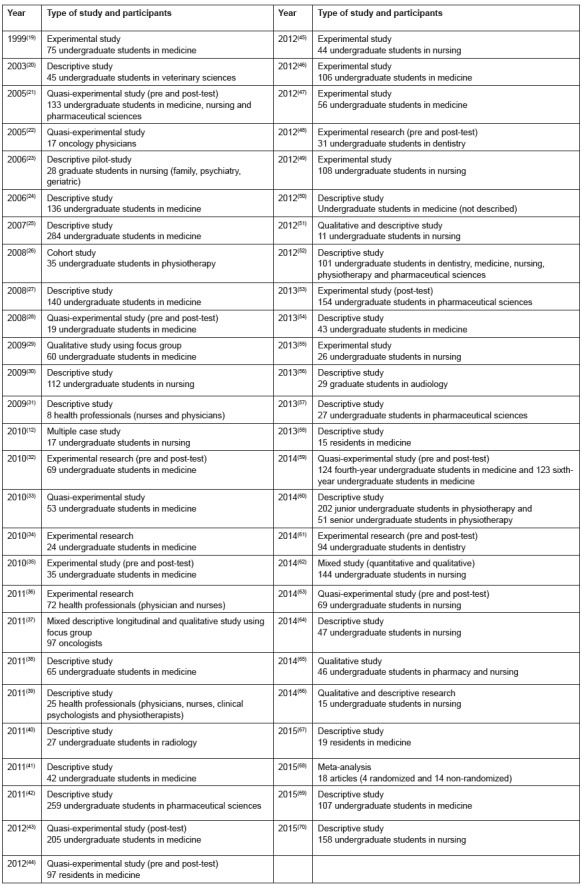
Method, year of publication, type and number of participants, 2016

As demonstrated in Figure 1, as regards the method used, among the studies analyzed, 23 were descriptive (43.4%), 13 experimental (24.5%), 8 quasi-experimental (15.1%), 4 qualitative (7.5%), 2 mixed (3.8%), 1 cohort (1.9%), 1 multiple case study (1.9%) and 1 (1.9%) meta-analysis. The year of publication, type and number of participants have been described in Figure 1.

## Discussion

Simulation has turned into a fundamental tool for the education and recycling of health professionals. It permits modeling clinical events in a safe environment, resulting in learning gains due to the possibility for the student to develop competencies, critical reasoning, decision making, teamwork and, mainly, to contribute to the strengthening of self-confidence^5^ ^-^ ^8^.

Simulation with dramatization resources has been used as a teaching strategy through clinical simulation, in the education of future professionals as well as in the training of active ones. When applied as such, it is able to offer the students the possibility to train skills and even competencies at a reasonable cost, in a safe environment, through the creation of scenarios with a wide range of complexities. In addition, it realistically reproduces an encounter with the (simulated) patient, which can strongly contribute to the learning objectives outlined^71^. It also offers the possibility of feedback by the simulated patient, which contributes and enriches the teaching-learning process^55^.

**Figure 2:**
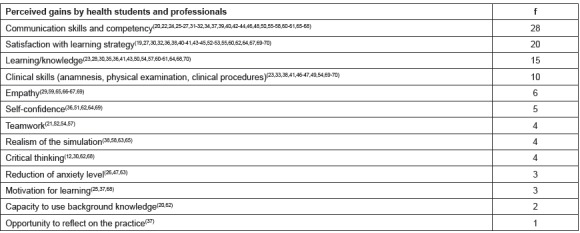
Perceived gains by students and professionals using dramatization resources and frequency, 2016

This study was aimed at identifying the gains health students and professionals perceived in clinical simulation using dramatization resources. Although the grey literature was not included, which can be considered a limiting factor, a large number of studies could be identified, observing that simulation with dramatization resources has been used expressive and effectively in the teaching and training process of health professionals in a wide range of scientific areas, also aiming to develop interprofessional competencies (Figure 1 and Figure 2).

*More than one perceived gain per article.

The dramatization strategy used needs to support the learning objectives of the activity. Different dramatization strategies were employed in the studies assessed; among these, the use of the simulated patient and role play stood out.

The simulated patient participates actively in the activity and, in the debriefing process, permits interactivity in the learner’s reflection. In addition, the patient needs to be engaged in the assessment of the activity. The use of the role play strategy allows the learner to empathetically experience the role of the patient, relative and/or of another professional, in an active, involving and dynamic manner, supporting the construction process of clinical competencies and effective communication. Clinical competence is a fundamental quality for professionals who are apt and capable of delivering high-quality care. The use of simulation can be considered an admirable tool for the students to find their action sphere, autonomy, adaptation and flexibility in the course of their development, in different realities^72^.

Among the gains identified in the studies analyzed, the enhancement of knowledge, development of empathy, of communication skills, satisfaction with the teaching-learning process, self-confidence, realism, reduction of the anxiety level, comfort, motivation to learn, capacity to reflect and think critically and teamwork skills were observed.

Communication was the gain that stood out in the studies analyzed. Health educators have been increasingly concerned with the inclusion of teaching-learning strategies for the development of communication skills, as effective communication is an essential clinical competency for the practice of health professions. It can be taught and qualified effectively by means of dramatization in simulated practices^20^ ^-^ ^21^ ^,^ ^25^ ^-^ ^27^ ^,^ ^29^ ^,^ ^31^. In the selected sample, the following dramatization strategies were widely used to develop communication: role play and simulated patient, mainly in situations that were difficult for the professionals to cope with, such as ethical dilemmas, communication of bad news, conflicts in the interprofessional team, among others^22^ ^,^ ^25^ ^,^ ^27^ ^,^ ^31^ ^,^ ^65^ ^-^ ^66^.

Satisfaction with the clinical simulation method has been increasingly valued at health institutions and is related to the motivation process for learning^30^. It is an indicator of best practices in the teaching-learning process and of good work conditions for the educators. It can be influenced by the desire and experience of the teaching staff. In the studies analyzed, the use of simulated patients and the realism of the strategy were the main indicators of this perceived gain^45^ ^,^ ^73^.

The realism benefits the activity and makes it successful, as it makes the participants consider the strategy as legitimate and authentic^32^ ^,^ ^38^ ^,^ ^58^ ^,^ ^63^ ^,^ ^65^ ^,^ ^74^. During the simulation, the realism can be translated by the fidelity of the simulated experience in approaching the actual environment. High-fidelity simulation approaches the practice with patients as closely as possible^75^. In the sample, the studies analyzed demonstrated that the learners perceived the use of the simulated patient as very close to the real patients. In addition, the following also contributed to the realism: the extent to which the environment approaches the facilities in practice, as well as the educators’ knowledge and preparation to trigger the emotions^19^ ^,^ ^58^ ^,^ ^63^ ^,^ ^65^ ^,^ ^76^. An environment close to the reality provokes the same psychological reactions in the individuals as they would have in practice, which makes the learners develop critical thinking and the decision-making skills required in an actual clinical scenario^5^ ^,^ ^77^ ^-^ ^78^.

What the teaching-learning process, knowledge and critical thinking are concerned, simulation with dramatization showed to be an innovative and diversified teaching-learning tool, which promotes the students’ opportunities to reflect on the practice^37^, strengthen the background knowledge^22^ ^,^ ^35^ ^,^ ^41^ ^,^ ^50^ ^,^ ^54^ ^,^ ^56^, understand the strong and weak points of their learning^60^, develop critical thinking^37^ ^,^ ^62^ and the opportunity to use previously acquired knowledge and skills^62^ and, therefore, enhances the awareness on the students’ actual capacities. In the studies observed, role play showed to be an interesting tool in the teaching-learning process^25^, in view of the learners’ level of acceptance^32^, as it makes the theoretical and practical knowledge significant, integrates and transforms it at the individual and collective levels^21^ ^,^ ^28^. It is also important to highlight that the simulated practices permit measuring and assessing the results obtained through instruments and/or video recordings for future clarifications^58^.

The studies also demonstrated that the simulations made the learners more trusting, minimizing the fear to undertake the procedures with the patients^20^ ^,^ ^26^ ^,^ ^30^_**,**_ mainly in the physical examination and communication processes^33^ ^,^ ^36^ ^,^ ^41^ ^,^ ^49^ ^,^ ^54^. Self-confidence also leads to the reduction of the anxiety level^26^ ^,^ ^44^ ^,^ ^47^ ^,^ ^63^ and increased comfort^44^ ^,^ ^47^.

Anxiety is a natural reaction, produced in response to certain situations in which the person needs adaptive resources. When confronted with critical activities for which they do not feel prepared, the learners report anxiety, tension, mainly when the care targets children and patients in severe and/or terminal conditions^79^. The stress and anxiety can negatively contribute and interfere in the teaching-learning process. The two main sources of anxiety in clinical practice are lack of knowledge and lack of skills^79^.

In the gains the learners perceived, the development of empathy could be observed, which involves the feeling of sensitization for the changes the other person feels and reflects moment by moment^80^. Empathy was a gain perceived in some studies analyzed^29^ ^,^ ^59^ ^,^ ^66^ ^-^ ^67^ ^,^ ^69^ and measured during the role play strategy^69^.

It is important to highlight that, in technical competency development, the dramatization comes with some limitations, as not all procedures can be executed on the simulated patients. To solve that difficulty, sometimes, mixed patients are used, like when a pelvis is attached to the simulated patient during urinary catheterization. In the sample of this review, it could be identified that simulation with dramatization was used in anamnesis ^46^, physical examination^19^ ^,^ ^38^, pelvic examination^23^ ^,^ ^33^ ^,^ ^47^ ^,^ ^49^ and postoperative pain assessment skills^36^. It was also observed that dramatization was used to develop critical thinking in punctual^30^ ^,^ ^35^ ^,^ ^62^ ^,^ ^68^ ^)^ studies, perhaps due to the fact that the physiological outcomes cannot be controlled in simulated patients.

## Conclusion

The large number of studies found in this research demonstrates that simulation with dramatization is a tool in the teaching-learning process, largely used in the education and qualification of health professionals.

In this process, in a wide range of health areas and also involving different professionals, different gains are obtained, among which satisfaction, self-confidence, knowledge, empathy, realism, reduced anxiety, comfort, communication, motivation, capacity to reflect and think critically and teamwork stand out. The evidences demonstrate the great possibility to use dramatization in the clinical simulation context.
